# Access challenges for patients with limited English proficiency: a secret-shopper study of in-person and telehealth behavioral health services in California safety-net clinics

**DOI:** 10.1093/haschl/qxad033

**Published:** 2023-08-14

**Authors:** Lori Uscher-Pines, Kandice Kapinos, Claudia Rodriguez, Samantha Pérez-Dávila, Pushpa Raja, Jorge A Rodriguez, Maya Rabinowitz, Mara Youdelman, Jessica L Sousa

**Affiliations:** RAND Corporation, Arlington, VA 22202, United States; RAND Corporation, Arlington, VA 22202, United States; RAND Corporation, Santa Monica, CA 90401, United States; RAND Corporation, Santa Monica, CA 90401, United States; University of California Los Angeles, Psychiatry and Behavioral Sciences, Los Angeles, CA 90073, United States; Brigham and Women's Hospital, Department of Medicine, Boston, MA 02120, United States; RAND Corporation, Boston, MA 02116, United States; National Health Law Program, Washington, DC 20005, United States; RAND Corporation, Boston, MA 02116, United States

**Keywords:** limited English proficiency, language assistance, telehealth, health centers, safety net, health equity

## Abstract

The recent growth of telehealth may be impacting access to care for patients, including those with limited English proficiency (LEP). Using a secret-shopper design, simulated patients contacted 386 safety-net clinics in California in both Spanish and English from February–March 2023. Callers stated that they were new patients seeking medication for depression, and they documented time to an appointment and available visit modalities (telehealth and in-person). Multinomial logistic regression models examined associations between clinic characteristics and available modalities. English-speaking callers were more likely to speak with a live scheduler and to obtain appointment information from a scheduler who could engage with them in their preferred language. Among Spanish-speaking callers who reached a live scheduler, 22% reached someone who did not engage (eg, were hung up on) and, as a result, could not obtain appointment information. The mean estimated time to a prescribing visit was 36 days and did not differ by language. Sixty-four percent of clinics offered both telehealth and in-person visits, 14% only offered in-person visits, and 22% only offered telehealth visits. More attention and resources are needed to support patients with LEP at the point of scheduling and to ensure choice of visit modality for all patient populations.

## Introduction

Fourteen percent of the US population speak Spanish at home, and 16 million Spanish speakers have limited English proficiency (LEP).^1^ Health care organizations receiving federal funding, including Medicare and Medicaid, are required by law to provide meaningful access to patients with LEP^2^; however, Spanish-speaking patients with LEP face numerous barriers in navigating the health care system, including when scheduling appointments.^3–5^ Compared with English-proficient patients, patients with LEP receive fewer health care services, have greater unmet mental health needs, and receive poorer quality care.^6,7^ The challenges start well before patients with LEP interact with the health care system: patients with LEP with a mental health condition are less likely to perceive a need for treatment or seek specialty behavioral health treatment.^8^ Recent studies have identified factors contributing to inequities in access and outcomes for Spanish-speaking patients, including lack of language services, perceived discrimination, and mistrust and privacy concerns.^6,9,10^ Disparities are exacerbated when patients with LEP lack access to language-concordant clinicians or qualified interpreters.^11^

With the growth of telehealth at the start of the COVID-19 pandemic, patients with LEP faced additional challenges accessing care. Organizations documented difficulties in bringing third-party interpreters into telehealth workflows, translating written materials to support telehealth visits, and serving patients with low digital literacy.^9,12,13^ Not surprisingly, numerous studies have shown that patients with LEP utilized telehealth at lower rates.^14–16^ It follows that, if clinics only offered telehealth visits, patients with LEP may have been more likely to forgo care.

After the spring of 2020, telehealth use began to decline in many clinical areas.^17^ Yet, it remained widespread for behavioral health care in safety-net settings.^18–20^ While telehealth visits represented 0% of all specialty behavioral health visits in California's Federally Qualified Health Centers (FQHCs) prior to the pandemic, in the summer of 2022, 62% of visits were delivered via telehealth.^21^ To date, we know very little about how this dramatic and enduring change in behavioral health care delivery has impacted the availability of different types of visits, wait times, and general experiences of patients with LEP in accessing care. Further, although many patients with LEP prefer in-person care,^22^ it is unclear whether they are offered a choice of visit modality.

We conducted a secret-shopper (audit) study where researchers posed as Spanish- and English-speaking Medicaid beneficiaries seeking medication treatment for depression. Although audit studies have been used for many years to explore access barriers for underserved patients,^23–25^ few have assessed how the interactions and experiences of Spanish-speaking callers differ from those of English speakers. Further, no studies have applied an audit methodology to understand how the introduction of telehealth and hybrid care models has affected access and care-seeking experiences. Our primary aim was to assess differences in the experiences of Spanish- and English-speaking Medicaid beneficiaries in obtaining care from behavioral health clinicians in safety-net settings. We also set out to explore whether wait times and modality options differed based on language.

## Data and methods

Data were collected using an audit methodology.^26^ In contrast to surveys, which may be biased in important ways (eg, recall bias, social desirability bias), audit studies can reveal real-world behavior and more effectively isolate the influence of particular factors (eg, LEP).

### Sample selection

We searched online health care provider directories of Medicaid managed-care plans in California to identify organizations that provided outpatient behavioral health services and served patients with Medicaid insurance. While we reviewed the public directories of all Medicaid managed-care plans (which insure 11 million low-income patients across 58 counties),^27^ we queried the 18 that had search capabilities that allowed us to filter by some of our inclusion criteria. To be included, an organization had to be (1) a hospital or non–hospital-based group practice, community mental health center, or FQHC that offered medication treatment delivered by specialty behavioral health clinicians (eg, psychiatrists, psychiatric nurse practitioners) and (2) treat adults. Organizations that only served specific populations (eg, HIV-positive, veterans) or indicated that they were not taking new patients in 1 or more directories were excluded. We also excluded FQHCs that only provided behavioral health services within primary care. This exclusion was important to ensure that analyses compared wait times and modality options for visits with specialty behavioral health clinicians. This process yielded 708 unique organizations (hereafter, “clinics”), which we randomly sampled using simple randomization after stratifying by region and clinic type.

### Data collectors and script

Three female data collectors or “callers” (C.R., S.P.-D., M.R.) posed as a new patient seeking medication treatment for moderate depression. Two callers were native Spanish speakers. We selected depression because it is one of the most common behavioral health conditions and, absent a crisis, would not signal a sense of urgency to schedulers. In California, patients with serious mental illness are treated within a separate system of care, so we intentionally designed a scenario that would be treated through standard outpatient care.

Callers followed a detailed script for each call (Supplement). Spanish and English scripts were identical, with 2 exceptions: Spanish and English callers used different names (if asked) and the Spanish script included additional questions about language assistance. At the start of each call, the caller asked if she could make an appointment as a new patient to get medication treatment for depression. Callers did not proactively offer additional information but did answer any questions that the scheduler asked (as specified in the script). If asked about health insurance, the caller mentioned a Medicaid managed-care plan applicable to the county where that clinic was located. The 2 Spanish-speaking callers collaborated to translate the English script into Spanish.

Because gatekeeping (ie, required steps before a patient can have a visit with a prescriber) is common for behavioral health services in safety-net settings, we tested the feasibility of the script and protocol with 45 test calls. For example, we wanted to verify that the caller could get schedulers to disclose wait times for prescribing visits even when multiple gatekeeping steps were required. We began our test calls with a draft script that the study team developed following review of published secret-shopper protocols. During the pilot testing process, we continued to refine our script in weekly team meetings (eg, prioritizing the collection of certain data given time constraints and adding answers to unanticipated questions from the scheduler). One significant change we made was to ask the scheduler to explain the process for getting care at the clinic assuming all gatekeeping steps were completed. No data from test calls were included in this analysis.

### Data collection

From February to March 2023, each sampled clinic was called by 2 different callers in Spanish and English within a 14-day period. Half of the clinics were randomly assigned to receive their first call in Spanish and the other half in English to mitigate against potential ordering effects. A caller would contact each clinic up to 2 times over the course of 1 week and left voice messages requesting a call back when they could not reach a live person. Callers abandoned calls if they had to wait on hold for longer than 10 minutes.

Using a standardized data-collection tool, callers documented (1) questions asked by the scheduler, (2) details on gatekeeping, (3) time (in calendar days) to the first available appointment with a prescriber (including time for any gatekeeping steps), (4) visit modalities available for prescribing visits (telehealth, in-person, both), and (5) language services for telehealth and in-person visits (ie, a bilingual clinician, a co-located staff person who would interpret, or a third-party interpreter) (Spanish only). Callers also documented general observations and impressions of each call (eg, time on hold, helpfulness of scheduler, Spanish proficiency of the scheduler). No appointments were made.

This study was declared exempt by RAND's Institutional Review Board and consent was not required. This study followed STROBE (Strengthening the Reporting of Observational Studies in Epidemiology) reporting guidelines.^28^

### Measures

Our analytic dataset included call details as well as clinic-level characteristics (eg, clinic type, zip code). We used clinic zip code to merge in county-level measures obtained from 2022 County Health Rankings.^29^ These measures included the following: mental health provider supply (county population divided by the number of mental health providers), proportion of the county population that is Hispanic/Latinx, and county urbanicity (defined using rural-urban continuum codes).

We measured access and clinic capabilities in several ways. First, we created a categorical measure of the status of each call to distinguish different reasons that callers were unable to obtain appointment information (“Call status” in Table 1). A key outcome was whether the caller could get to the point where they could obtain appointment information (eg, available visit modalities). To reach this point, a caller would need to reach a live scheduler who was willing and able to engage them in their preferred language. Getting to the point where the caller could obtain appointment information is necessary, but not sufficient, to actually obtaining an appointment. This is the case because quoted wait times could be very long or the ability to schedule an actual appointment could depend on the outcome of 1 or more gatekeeping steps.

**Table 1.** Characteristics of calls by language of simulated patient (unadjusted).

	Language of simulated patient				
	English		Spanish		
	n	%	n	%	*P*
Call status (239 clinics)					
Unable to speak to live scheduler	24	10	42	18	.02
Hung up on or told no one at clinic could communicate in Spanish	0	0	43	18	.00
Ineligible	15	6	9	4	.22
Not taking new patients	51	21	48	20	.73
Able to connect and obtain appointment details	149	62	97	41	.00
Call details (173 clinics/246 calls)					
Any gatekeeping (yes/no)	95	64	68	70	.30
Require intake visit (yes/no)	78	52	57	59	.32
Asked about insurance(yes/no)	74	50	59	61	.09
Put on hold (yes/no)	90	60	81	84	.00
On hold >5 minutes (yes/no)	41	28	47	48	.00
Modality options (173 clinics/246 calls)					
In-person visits offered	72	49	84	86	.00
Telehealth visits offered	99	66	83	86	.00
Both types of visits offered	72	48	69	71	.00

The table shows how call status, call details, and modality options differed by the language of the caller. Source: Authors’ analysis of call data. Sample for rows 1–6 includes calls in both languages to 239 clinics; samples for rows 7–16 include only calls where the simulated patient was able to get to the point in the scheduling process where they could obtain appointment details (246 calls/173 clinics). Call status categories are mutually exclusive.

Second, in cases where callers could obtain appointment information, we examined several processes and clinic capabilities: whether there was gatekeeping of any kind, whether an intake visit was required, whether and how long the caller was placed on hold, wait time to obtain a visit with a specialty behavioral health clinician, and visit modalities for prescribing visits. It should be noted that, although we present differences in processes and clinic capabilities by language, these results should be interpreted with caution because Spanish-speaking callers were much less likely to reach a scheduler who could provide this information due to access challenges. In particular, if only clinics most equipped to serve Spanish-speaking patients provided appointment details, any comparisons across language would be biased. Because of this issue and the fact that the availability of different visit modalities was a key research question, we also assessed available visit modalities at the clinic level. A clinic was categorized as offering a particular modality if its scheduler told either the Spanish- or English-speaking caller that the modality was available.

### Analysis

We used standard chi-square, *t*, and Mann-Whitney tests to examine differences in proportions and means and medians by language of the caller. We also estimated multinomial logistic regression models to examine the associations between clinic characteristics and available visit modalities. Statistical analysis was performed using Stata 17.0 MP (StataCorp). *P* < .05 was considered statistically significant, and all tests were 2-tailed.

### Limitations

There are several limitations. First, we only contacted clinics in California. California has the country's largest population of Spanish speakers with LEP and the most state laws related to language access.^30^ Access challenges may be worse in other states. Second, we only evaluated the experiences of Spanish speakers, and findings may not be generalizable to other patients with LEP. Third, we only explored the process of obtaining a new patient appointment via phone. Additional disparities may become evident when scheduling appointments online or in subsequent stages of treatment-seeking. Fourth, wait times should be considered estimates because many clinics had gatekeeping requirements, and the wait time might depend on the outcome of the gatekeeping steps. Further, in some FQHCs, primary care and behavioral health are different departments, and the scheduler may not have had visibility on times within behavioral health. Fifth, we did not have information on clinic practice size. Finally, callers did not formally evaluate the Spanish proficiency of the scheduler using a validated assessment tool.

## Results

**Figure 1.**
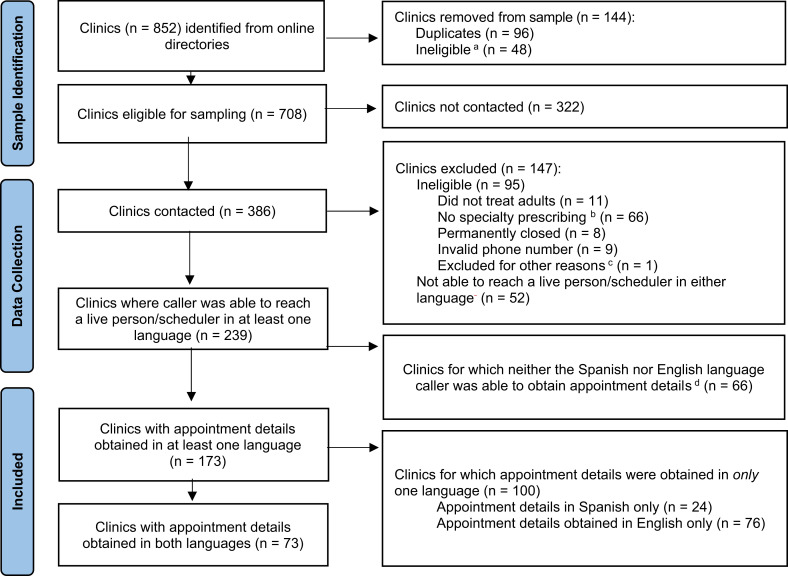
Flow diagram showing how the sample of clinics was constructed, including reasons for exclusions (n = 852). Source: Authors’ analysis of call data. ^a^We excluded Veterans Affairs and Indian Health Service clinics, telehealth-only providers, and other organizations that served specific populations (eg, homeless individuals; those living with HIV/AIDS). ^b^Excluded organizations included group practices that were not providing medication treatment (n = 36), organizations whose behavioral health services were limited to intensive outpatient or emergency care only (n = 12), and Federally Qualified Health Centers (FQHCs) that provided behavioral health prescribing within primary care only (n = 17). ^c^One clinic was reached by 2 separate callers in the same language due to an administrative error, and thus was excluded from all analyses. ^d^Reasons for not being able to obtain appointment details varied depending on the language of the caller. For Spanish-language callers, reasons included not taking new patients (n = 38), being hung up on or told no Spanish speaker at clinic (n = 20), not able to connect with a person (n = 7), and clinic ineligibility due to no specialty outpatient behavioral health prescribing (n = 1). For English-language callers, reasons included not taking new patients (n = 44), not able to connect with a person (n = 13), and clinic ineligibility due to no specialty outpatient behavioral health prescribing (n = 9).

A total of 386 clinics were contacted. Among these, 95 (25%) were ineligible (eg, did not provide medication treatment). Further, for 52 clinics (14%), neither caller was able to speak to a live scheduler. For 239 clinics (62%), callers were able to reach a scheduler in 1 or both languages (Figure 1). These clinics included 163 (68%) specialty behavioral health clinics and 76 (31%) FQHCs.

Among the 239 clinics reached in 1 or both languages, English-speaking callers were more likely to speak with a scheduler (ie, reach a live person; 90% vs 72%; *P* = .02) and more likely to reach the point in the scheduling process where they could obtain appointment information in their preferred language (62% vs 41%; *P* < .01) than Spanish-speaking callers (Table 1). Among Spanish-speaking callers who spoke with a scheduler, 43 (22%) reached someone who would not engage with them because of lack of language assistance (eg, were hung up on, were told that no one on the staff could speak with them in Spanish). In contrast, English-speaking callers who spoke with a scheduler were never hung up on in mid-conversation or told that staff could not speak with them in their preferred language. In 1 example of a failed Spanish-language call, a Spanish-speaking caller reached an English-speaking scheduler at a group practice in Los Angeles. When the caller asked (in Spanish) if the clinic was taking new patients, the scheduler repeated several times (in English) that they did not speak Spanish. When asked if someone else at the clinic spoke Spanish, the scheduler said “no” and hung up.

There were 246 calls (149 in English and 97 in Spanish) across 173 clinics where appointment details could be obtained. In these cases, callers successfully spoke with a language-concordant scheduler or reached a scheduler who brought in an interpreter to discuss scheduling (Spanish only). For 21 (14%) of Spanish-speaking calls, callers observed that the scheduler with whom they interacted was not proficient in Spanish (eg, seemed unsure about word choice, often reverting to English).

The large majority of callers who obtained appointment details (64% of Spanish- and 70% of English-speaking callers) were subject to gatekeeping. There were no statistically significant differences in whether the clinic had any gatekeeping or required an intake visit (the most common type of gatekeeping) by language (*P* = .30). Spanish-speaking callers were marginally more likely to be asked about their insurance status relative to English-speaking callers (61% vs 50%; *P* = .09). Spanish-speaking callers were also more likely to be placed on hold for 5 or more minutes (48% vs 28%; *P* = .001). However, Spanish-speaking callers were significantly more likely to be told that they had a choice of visit modalities (71% vs 48%; *P* < .01). The mean wait time to a prescribing visit across all callers was more than 1 month (36.18 days). The wait time was not significantly different for Spanish and English speakers (*P* = .50) (Table 2).

**Table 2.** Time to prescribing visit, by language (unadjusted).

	Calls with appointment information (n = 246)	Language of simulated patient caller				
		English		Spanish		
		Statistic/%	n	Statistic/%	n	*P*
Total time to visit						
Mean days	36.2	37.6	120	33.7	70	.50
Median days	28	28	120	30	70	.44
Percentage of calls without estimate	22%	19.5%	29	27.8%	27	.05

The table shows mean and median days to a visit with a prescriber and differences by language of caller. Source: Authors’ analysis of call data. Time to prescribing visit accounts for any required gatekeeping steps (eg, time to intake + time to visit with specialty behavioral health clinician after the intake.) The *P* values for difference across languages were obtained from chi-square, *t*, and Mann-Whitney tests to compare proportions, means, and medians, respectively. Mean and median values were obtained only from calls with nonmissing values (sample sizes shown in “n”). The % of calls where the wait time was unknown are calculated as a fraction of completed calls (for which we have appointment details) within each language group (eg, 149 English and 97 Spanish). In some cases, the scheduler could not provide an estimate because the wait time would depend on the outcome of a gatekeeping step, or the scheduler would not provide a wait time estimate until the patient was assigned to the clinic and had completed required paperwork.

**Figure 2.**
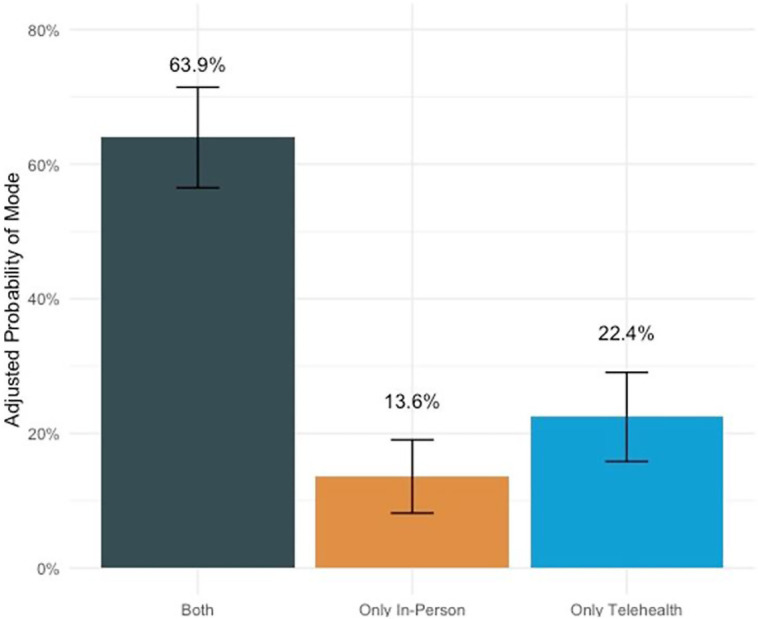
Adjusted probability of offering different visit modalities (n = 147 clinics). This figure shows the adjusted proportion of clinics that only offer telehealth visits, only offer in-person visits, and offer both visit types. Source: Authors’ analysis of call data. These probabilities were obtained post-estimation from a multinomial logistic regression where the mutually exclusive categorization of visit modalities (only in person offered, only telehealth offered, or both offered) was regressed on an indicator for whether the clinic was an Federally Qualified Health Center (FQHC; yes/no), whether the county had a majority >50% Latinx population, whether the county was in a metropolitan area (defined as “rural-urban continuum code” of 1–3), and the population (in 100’s) in the county divided by the number of mental health providers. Bars represent 95% CIs.

Adjusted analyses showed that 64% of clinics offered both telehealth and in-person visits, 14% offered only offered in-person visits, and 22% offered only telehealth visits. We did not identify any statistically significant differences in these probabilities by clinic-level factors (eg, clinic type), but this may be due to lack of statistical power (Figure 2).

**Figure 3.**
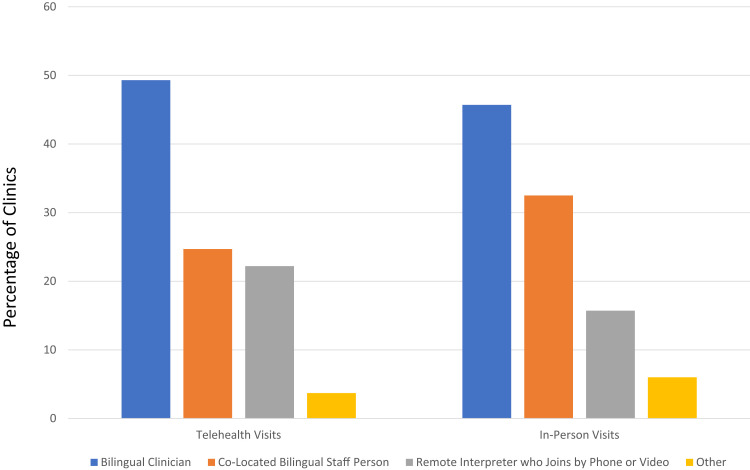
The figure shows the types of language assistance available for Spanish speakers in telehealth and in-person visits. Source: Authors’ analysis of call data. Data for 83 clinics that reported offering in-person visits and 82 clinics that reported offering telehealth visits to Spanish-speaking callers. “Other” includes using translation software or requesting that the patient have a friend of family member join the visit who could interpret on their behalf.

Spanish-speaking callers inquired about which language-assistance services were typically provided for in-person and telehealth visits. Language-concordant care with a Spanish-speaking prescriber was provided at 40 (50%) clinics for telehealth visits vs 38 (46%) for in-person visits (Figure 3).

## Discussion

Spanish-speaking callers in our study had more challenges connecting with a scheduler and getting to the point in the scheduling process where they could obtain appointment information. This is important because obtaining appointment information is a required step in successfully scheduling an appointment.

We observed evidence of potential discrimination during the scheduling process, with nearly 1 in 5 Spanish calls ending in the scheduler hanging up on the caller or informing them that no one was available to assist them in Spanish. In cases where Spanish-speaking callers could obtain appointment information, they were not subject to longer waits or fewer modality options. However, wait times to a prescribing visit (mean of 36 days) were long for all simulated patients, and well beyond established targets.^31^ Further, approximately one-fourth of clinics in the sample did not offer in-person care and one-third did not offer both modalities, suggesting that more work is needed to ensure that patients can receive their preferred treatment modality.

There are only a handful of secret-shopper studies that have described the process of obtaining appointments among Spanish-speaking patients seeking care and, to date, no published studies have focused on behavioral health services.^32–37^ Further, approximately half of such studies that we identified had researchers call in English on behalf of a Spanish-speaking family member,^32,34^ and thus did not evaluate meaningful access to care. One secret-shopper study by Azua et al^33^ that described access to outpatient orthopedic appointments had similar findings: Spanish speakers were less likely to secure appointments due to challenges with reaching schedulers, but wait times to an appointment did not differ by language. Our findings are consistent with multiple studies demonstrating access barriers faced by patients with LEP that may contribute to underutilization of care and negative health outcomes.^4,6,38^ Further, our finding that 38% of clinics listed in online provider directories could not be reached by any of our callers or were not taking new patients provides further evidence of the existence of “ghost networks” (ie, providers who are listed by payers as in-network options but are nonexistent or unavailable) in behavioral health.^39^

Our findings reveal that additional work is needed to implement and enforce guidelines on linguistically appropriate care (eg, Title VI of the Civil Rights Act of 1964 and Section 1557 of the Patient Protection and Affordable Care Act).^40,41^ Specifically, more attention and resources are needed to support patients with LEP at the critical point of scheduling. Although patients with LEP have a right to receive meaningful access in all interactions with health care organizations, existing guidance tends to focus on patient–clinician communication and critical documents related to the medical visit, and seldom addresses interactions between patients and schedulers.^42–45^

What can health care organizations do to reduce inequities in access to behavioral health care for patients with LEP who are seeking care? In areas with large numbers of Spanish speakers, organizations can prioritize the hiring of bilingual staff in key positions such as receptionists and schedulers and promote high-quality medical language courses. They can also simplify the workflow required to bring interpreters into scheduling discussions and improve training on these workflows. Further, health care organizations can implement, and in some cases encourage, the use of online scheduling systems designed to meet the digital and linguistic needs of patients. Such efforts could be supplemented through the provision of interpretation or care navigation services at the county, regional, or state level (eg, hotline for patients with LEP that connects them to interpreters who can then join scheduling calls at individual clinics). It is important to recognize that there is a cost to providing language assistance in every interaction, and laws requiring meaningful access are commonly criticized as unfunded mandates.^46^ Hiring multilingual front office staff may not be a feasible solution to communication barriers in safety-net settings where many languages are spoken and workforce shortages are common. Broader efforts at the payer or state level, however, could generate economies of scale.

Interestingly, many clinics in our sample had Spanish-speaking clinicians (∼50%). Other secret-shopper studies have shown that only 9–43% of visits with Spanish-speaking patients would be delivered by Spanish-speaking clinicians.^32–34^ Nonetheless, one might expect that telehealth would provide opportunities to bring in language-concordant clinicians from outside the community. The fact that the availability of Spanish-speaking clinicians was so similar for in-person and telehealth visits in our study represents a missed opportunity to fully leverage the advantages of telehealth. Telehealth may also offer other advantages for patients with LEP that require further study. For example, telehealth platforms can include features to improve communication (eg, closed captioning) and allow for participation by family members who are not in the same location.

Our results suggest that a substantial fraction of safety-net clinics do not offer in-person care and as many as one-third do not give patients a choice of visit modalities. Numerous organizations will need to make changes to their services to be compliant with California's new law requiring that clinicians who offer telehealth also provide in-person options or refer to in-person clinicians.^47^ Payers and policymakers should take steps to ensure patient choice because limited in-person availability in some communities should not become another barrier to patients receiving the behavioral health services they need or prefer.

In conclusion, this study highlights the significant challenges faced by Spanish-speaking individuals with LEP in accessing behavioral health services and the need to ensure access to both telehealth and in-person visits for all patient populations. Further research is needed to explore inequities in access in other states and stages of the care-seeking process, as well as to assess the efficacy of potential interventions to overcome access challenges.

## Contribution statement

L.U.-P. had full access to all the data in the study and takes responsibility for the integrity of the data and the accuracy of the data analysis.

## Supplementary Material

qxad033_Supplementary_Data
