# Supplementary material for: Access challenges for patients with limited English proficiency: a secret-shopper study of in-person and telehealth behavioral health services in California safety-net clinics
Source: Health Aff Sch. 2023 Aug 14;1(3):qxad033. doi: 10.1093/haschl/qxad033 (PMC10986294; doi:10.1093/haschl/qxad033)
Supplement: qxad033_Supplementary_Data [file qxad033_Supplementary_Data.zip › appendix.docx]

# Supplement: Spanish and English Scripts

## [Español]- Depresión

**Si el número está incorrecto:**

Número fuera de servicio (Registra Sí/No)

**Si es correo de voz:**

[Dejar Mensaje] “Hola, mi nombre es Fernanda Gómez. Soy una paciente nueva y quiero hacer una cita con alguien que me pueda recetar medicamentos para la depresión. ¿Puede pedirle alguien que trabaje ahí que hable español que me llame al xxx-xxx-xxxx?”

[Registra si hay un menú de opciones en Español (sí/no]

**Si contestan en persona:**

**Apertura (si recepcionista contesta en inglés):** Hola, ¿hablas español? No inglés ¿Puedo hablar con alguien que hable español? (Registre lo que hace la persona si no habla español: 1) ofrecer intérprete; 2) ofrecer devolver la llamada; 3) colgar; 4) ponerte en espera para llamar a alguien del personal que hable español)

**Apertura (si recepcionista habla español):** “Hola, mi nombre es Fernanda Gómez. Me gustaría hacer una cita con alguien que pueda recetar medicamentos para la depresión. Soy una paciente nueva. ¿Está recibiendo pacientes nuevos? (**Registra:** ¿Nuevos pacientes? Sí/No)

[Responde todas las preguntas que te hagan de acuerdo con los detalles a continuación. Registra las preguntas que te hicieron (p. ej., qué seguro tienes, si eres residente legal) antes de que la persona encargada determinara si estaban aceptando nuevos pacientes. (Registra la razón por la cual no están aceptando pacientes nuevos (p.ej., al máximo de su capacidad, no recetan medicamentos, no tratan adultos)]

**Si no aceptan pacientes nuevos:**

"¿Sabe a quién más en el área puedo llamar para una cita?" Necesito ver a un proveedor que hable español o que pueda traer a un intérprete. ¿Qué clase de oficina es esa? (Registra: Recomendación Sí/No; Detalles de la Recomendación)

**Si toma pacientes nuevos Y cumple con los criterios de inclusión.**

[La persona en recepción generalmente proporciona detalles sobre los pasos requeridos o hace preguntas adicionales. Registra las preguntas y contéstalas, documentando *el momento* de la conversación en el que las diferentes preguntas fueron hechas).]

“Gracias por describirme el proceso. Ahorita solo estoy tratando de averiguar cuál es el mejor lugar para tener atención el próximo año más o menos. Estoy viendo un par de lugares diferentes. ¿Puede responder algunas de mis preguntas para poder decidir mejor?”

Necesito una cita en español. ¿Tiene personal que pueda verme en español? ¿Como funciona? ¿El médico habla español o traerán un intérprete?

- [Si la visita de admisión se requiere antes de la visita para prescribir] Cuándo es la fecha más próxima para la cita de admisión?
  - **[OPCIONAL]** ¿Esa visita de admisión sería en persona, en línea, o cualquiera de las dos?
- Si todo sale bien con la visita de admisión, ¿cuánto tiempo hasta que vea a alguien que pueda recetarme medicamentos? ¿Podré obtener medicamentos en la primera visita? ¿Qué tipo de proveedor vería? Estoy buscando el primero disponible.
  - Para las visitas de medicación, ¿están disponibles en línea? ¿En persona? ¿Ambas?
  - ¿El idioma en el que atienden es diferente en línea que en persona? ¿Cómo son diferentes?
  - ¿Hay una diferencia en el tiempo de espera si elijo en video o en persona?
  - [Si telesalud se ofrece] **[OPCIONAL]** ¿Sus médicos hacen llamadas por video o por teléfono?

[En todo caso, escoge la opción con la disponibilidad más cercana cuando te den opciones]

“Muchas gracias por esos detalles. Espero ver a alguien un poco antes, así que volveré a llamar una vez que decida qué tiene más sentido para mí... O "Espero encontrar un lugar que pueda hacer "tanto en persona como en línea" o "pueda hacer video o en línea” O “pueden verme en persona”. O “Espero encontrar un médico bilingüe”. Volveré a llamar una vez que decida a dónde quiero ir.

**Si se hacen varias preguntas, responde pero desvía lo más rápido posible:**

-**Desvía preguntas:** “En realidad, ¿puedo obtener algunos detalles sobre cómo sería ser un paciente aquí para conseguir medicamentos para la depresión? Estoy tratando de decidir a dónde ir y saber cómo funcionan las cosas aquí me ayudará a decidir. [Ir a las preguntas principales]

-**Evita programar una cita:** "Oh, está bien, solo esperaba conseguir una cita un poco antes, así que creo que revisaré y te devolveré la llamada".

**Si se le informa sobre los pasos de control (por ejemplo, hablar con una enfermera, obtener una autorización previa)**

**-Evita tomar cualquier paso siguiente:** OK, gracias por explicarme el proceso…. Antes de transferirme…. lo que realmente quiero es entender cómo funcionaría recibir atención suponiendo que hice todas estas cosas/tuve todas estas cosas en su lugar. [Ir a las preguntas principales].

**Si se ofrece una cita (la primera disponible):**

-**Evita programar una cita:** “De acuerdo, déjame comprobar si puedo salir del trabajo ese día y te devolveré la llamada”

**No hagas una cita ni permitas que te reserven un espacio**

**Información de identificación para las preguntas**

**¿Nombre?** Fernanda Gomez

**¿Edad? 36**

**¿Fecha de nacimiento?** 4-14-86 [no des esta información si parece que la van a utilizar para crear una cita o un registro nuevo en su sistema]

**¿Número de teléfono?** “Probablemente saque un nuevo número muy pronto, por lo que no tiene sentido darle este.”

**¿Dirección?** “Um, estoy entre casas en este momento, me estoy quedando con un amigo, así que prefiero no dar mi dirección”. O Me gustaría esperar para dar mi dirección hasta que decida a dónde quiero ir para recibir atención.

**¿Es residente legal de EE. UU?** Sí

**Seguro médico**

**¿Cuál es su seguro médico?** “Tengo seguro de Medi-Cal [Medicaid] [nombre del plan de atención administrada dominante en el condado donde se encuentra la clínica]; aceptan ese?” (**Registra:** Acepta mi seguro Sí/No)

**¿Cuál es su número de seguro?** “Lo siento, no lo tengo conmigo en este momento. Puedo encontrarlo y devolverte la llamada.

**Ubicación**

**¿Dónde vive O qué ubicación prefiere?** “Estoy tratando de solucionar mi situación de vivienda permanente. Ahorita solo me quedo con un amigo mientras busco un lugar. Puedo llegar a cualquier ubicación en el condado O no tengo una gran preferencia. Tomaré cualquier ubicación que tenga la disponibilidad más cercana”.

**¿En qué alcaldía vive?** [Compara la alcaldía de la hoja de cálculo]

**Historial**

**¿Tiene un proveedor de atención primaria? ¿Proveedor de BH?** “Acabo de mudarme aquí desde el estado de Washington, así que todavía no tengo médicos”. No he visto a mi médico en el estado de Washington durante aproximadamente un año y no me atenderá desde otro estado.

**Cuénteme más sobre sus necesidades/lo que está buscando** “Tengo depresión y está empeorando. Tomé medicamentos hace un par de años y me ayudaron, pero tuve algunos efectos secundarios negativos, como cansancio y aumento de peso. Quiero ver si puedo probar con un medicamento diferente”

**Control**

**¿Le asignaron a esta clínica?** "Aún no. Solo quiero tener una idea de mis diferentes opciones en este momento. Pediré que me asignen si decido que tiene sentido venir aquí.”

## [English]- Depression

**If Wrong Number:**

-Number not in service (Record Yes/No)

**If Voice Mail:**

[Leave Message] “Hi, my name is Sophia Torres. I am a new patient and I want to make an appointment to get medication for depression. Can you call me back at xxx-xxx-xxxx?”

**If Live Person:**

**Opening:** “Hi, my name is Sophia Torres. I’d like to make an appointment with someone who can prescribe medication for depression. I am a new patient. Are you taking new patients? (**Record:** New Patients? Yes/No)

[Answer all questions that are asked according to details below. Record questions that were asked by the scheduler/intake person (e.g., what insurance do you have, are you a legal resident), indicating when in the conversation these questions were asked (e.g., before/after the scheduler told you whether they were accepting new patients). Record reason for not accepting new patients (e.g., at capacity, don’t do medication, don’t treat adults)]

**If not taking new patients:**

“Do you know who else in the area I can call for an appointment?”

What kind of office is that? **(Record:** Recommendation Yes/No; Details on Recommendation**)**

**If taking new patients AND meets inclusion criteria.**

[Scheduler usually provides details on the steps required or asks additional questions. Record questions asked and answer them, documenting *when* in the conversation different questions were asked).]

“Thanks for describing the process to me. Right now, I am just trying to figure out the best place for me to get care over the next year or so. I am looking at a couple different places. Can you just answer a few of my questions so I can better decide?”

- [If an intake visit is required before the prescribing visit] How long until the earliest available intake visit?
  - Would that intake visit be in-person, telehealth, either one?
- If everything goes well with the intake visit, how long until I would see someone who can prescribe depression medication? Do you know if I would be able to get medication at the first visit? What kind of provider would I see? I am looking for the first available.
  - For those medication visits, are they available via telehealth? In-person? Both?
  - Is there a difference in wait times if I want in-person vs. telehealth?
  - [If telehealth is offered] Do your providers do phone or video visits?

[In all cases, go with the earliest available when they give you a choice]

“Thanks so much for those details. I am hoping to see someone a bit sooner so I’ll call back once I decide what makes the most sense for me…OR “I am hoping to find a place that can do both in-person and telehealth” or “can do telehealth” OR “can see me in person.” I’ll call back once I decide where I want to go.

**If several questions are asked, answer but divert as quickly as possible:**

-**Divert Qs:** “Actually, can I just get some details about what it would be like to be a patient here for depression medication? I am trying to decide where to go and knowing how things work here will help me decide. [Go to core questions]

-**Avoid Booking:** “Oh OK, I was just hoping to get in a little sooner, so I think I’ll check around and give you a call back.”

**If told about gatekeeping steps (e.g., talk to a nurse, get pre-authorization)**

**-Avoid taking any steps:** OK, thanks for explaining the process…. Before you transfer me…. What I really want though is to understand how getting care would work, assuming I did all these things/had all these things in place. [Go to core questions]

**If appointment is offered (first available):**

-**Avoid Booking:** “OK, let me just check to see if I can get off from work that day and I’ll call you back”

**Do Not Make an Appointment or Let them Hold a Slot for You**

**Identifying Information for Questions**

**Name?** Sophia Torres

**Age?** 36

**Birthday?** 7-1-86 [do not provide this if it seems they’ll be using it to create an appointment or new record for you in their system]

**Phone Number?** “I may be getting a new number very soon so it probably doesn’t make sense to give you my current one.”

**Address?** “Um, I’m between houses right now – I’m staying with a friend so I’d rather not give my address.” OR “I’d like to wait to give my address until I decide where I want to go for care.”

**Are you a legal resident of the U.S?** Yes

**Health Insurance**

**What is your insurance?** “I have Medi-Cal [Medicaid] insurance [name dominant managed care plan in county where the clinic is located]; do you take that?” (**Record:** Accept my insurance Yes/No)

**What is your insurance number?** “Sorry, I don’t have that here. I can find it and call you back.”

**Location**

**Where do you live OR which location do you prefer?** “I am trying to figure out my permanent living situation. Right now, just staying with a friend as I look for a place. I can get to any location in the county OR no strong preference- I’ll take whatever location has the earliest availability.**”**

**What county do you live in?** [Share county listed on spreadsheet]

**Backstory**

**Do you have a primary care provider? BH provider?** “I just moved here from Oregon so I don’t have any doctors yet.” I haven’t seen my doctor in Oregon for about a year and she won’t treat me from a different state.

**Tell me more about your needs/what you are looking for?** “I am having depression and it is getting worse. I was on meds a couple years ago and they helped but I had some bad side effects like being tired and gaining weight. I want to see if I can try a different medication.”

**Gatekeeping**

**Have you been assigned to this clinic?** “Not yet. I just want to get a sense for my different options right now. I’ll ask to get assigned if I decide it makes sense to come here.”
